# Supplementary material for: High nutrient loads amplify carbon cycling across California and New York coastal wetlands but with ambiguous effects on marsh integrity and sustainability
Source: PLoS One. 2022 Sep 9;17(9):e0273260. doi: 10.1371/journal.pone.0273260 (PMC9462672; doi:10.1371/journal.pone.0273260)
Supplement: Attachment — Submitted filename: PONE-D-22-07419_review.pdf [file pone.0273260.s004.pdf]

High nutrient loads amplify carbon cycling across California and New York coastal wetlands but with ambiguous effects on marsh integrity and sustainability

PONE-D-22-07419

May 30, 2022

1. **Recommendation (Answer options: Accept, Minor Revision, Major Revision, Reject**
   Minor Revision

2. **Is the manuscript technically sound, and do the data support the conclusions? (Answer options: Yes, No, Partly)**
   Yes

3. **Has the statistical analysis been performed appropriately and rigorously? (Answer options: Yes, No, I don't know, N/A)**
   Yes

4. **Does the manuscript adhere to the PLOS Data Policy? Additional details can be found at http://journals.plos.org/plosone/s/materials-and-software-sharing. (Answer options: Yes, No)**
   Yes

5. **Is the manuscript presented in an intelligible fashion and written in standard English? (Answer options: Yes, No)**
   Yes

6. **Review Comments to the Author (minimum 200 characters)**

   General comments:
   The Authors have a well written manuscript that outlines a study that measured a suite of belowground characteristics across salt marshes of varied nutrient exposure. The Authors noted salt marsh "drowning" in areas of high nutrient exposure, which inspired this study to determine how high nutrient exposure may influence belowground characteristics that would ultimately lead to marsh loss. The Authors sampled two sites within four estuaries, two within New York and two within California, for a total of eight salt marsh sites that ranged a nutrient exposure gradient. The Authors measured factors such as litter breakdown, belowground productivity via in-growth bags, soil respiration and nutrient content, and soil microbial biomass. The Authors found sites with higher nutrient exposure had higher belowground productivity as well as faster organic matter breakdown. Due to these results, the Authors concluded that high nutrient exposure alone

could not be the cause of marsh drowning, but certainly could be a confounding factor due to the increased mineralization.

The Authors present a nicely put together manuscript. The study design, by incorporating estuaries of various nutrient exposure, cleverly uses the natural landscape to elucidate large trends to aid in our understanding of marsh stability. The data analyses and accompanying tables and figures are comprehensive and easily discernible. The writing is clean, clear, and concise.

There are a few items that should be addressed and/or expanded upon in the Discussion. In Line 472, there is mention that soil texture can influence nutrient impacts. It would be good to not only summarize the previous work that is referenced on how soil texture can influence nutrient impacts but also apply this information to the study results. The California and New York sites varied in soil texture (e.g., New York had much higher sand content) and since California and New York sites are not evenly distributed along the nutrient exposure gradient (e.g., California sites are the two highest nutrient exposure sites) the soil texture differences may have played a part in findings beyond just the nutrient exposure gradient. The Discussion would also benefit on some discussion of the differences between the two dominant plant types used in this study. In the Results, there is mention of *Salicornia pacifica* "woody nature" (Line 375-377), but this should be mentioned again in the Discussion, along with the differences in breakdown rates between succulent forb and graminoid leaves.

<u>Specific comments:</u>
Line 65-70: Consider adding more salt marsh related examples.

Line 82: Appears two spaces after the comma.

Line 85-86: Sentence is a bit awkwardly worded; consider revising.

Line 91-93: Briefly articulate how "decomposition is problematic…to wetland integrity."

Line 96: Consider revising or including an example of what is meant in terms of "soil integrity."

Line 102-105: Revise and make more concise.

Line 141-142: Suggest including the other two estuaries studied.

Line 145: Define what soil integrity means in the context of this study, especially as to differentiate from earlier mentioned marsh integrity, which soil integrity appears to one of its measures.

Line 166: What is exactly meant by “nutrient availability conditions”? Is this related to the “nutrient exposure” that is stated later?

Line 169-172: Would be nice to re-state which two are the eutrophic estuaries. Although it would appear that really one site in each of the two CA estuaries are eutrophic and two are not. This may need to be stated as from the Introduction, it would seem that the entire Elkhorn Slough estuary is considered eutrophic. Additionally, are there any signs of marsh drowning in the other two estuaries stated here, like the two mentioned in the Introduction?

Line 200: Data were not available

Line 231: State that live and dead material were not separated (information inferred from Line 510-511).

Line 238: How was material dried prior to deployment?

Line 245: What were the intervals that the bag collections occurred?

Line 260: Soil surface temperatures?

Line 311-313: Consider sentence revision as reads a bit confusing.

Line 322-325: Consider sentence revision.

Line 455: “Here” isn’t needed.

Line 466-469: Consider sentence revisions as reads a bit confusing.

Line 475-483: This paragraph acknowledges an important aspect and is appreciated. Perhaps here too can add the differences in nutrient ratios between succulent and graminoid leaves.

Line 518-522: This should be expanded a bit more and as just stating “substrate plays an extremely important role” doesn’t seem to fully flesh out why the results were not as expected.

Line 591-594: Suggest also including how these regional differences may help to understand impacts to belowground metrics.

Figure 4: It is unclear why New York and California sites are separated here, but the rest of the analyses compare all sites together along nutrient exposure gradient. In addition, the colors are a bit hard to see.

7. PLOS authors have the option to publish the peer review history of their article (https://journals.plos.org/plosone/s/editorial-and-peer-review-process#loc-peer-review-history). If published, this will include your full peer review and any attached files. Do you want your identity to be public for this peer review? (Answer options: Yes, No)
   No

   a. If you answered "Yes" above, that you would like your identity to be public, please provide your full name, as it should appear on the published peer review. Please do not sign the review on behalf of another person.

Confidential to Editor

1. **Do you have any potentially competing interests? If none, type "None." Our policy on competing interests can be found at http://journals.plos.org/plosone/s/competing-interests.**
   None

2. **Did you receive any assistance in preparing this review (e.g. from a post-doc or graduate student)? If yes, please provide their name and title. If you have chosen to reveal your identity to the authors in question 7 above, please include the name of the person who assisted you in the response to question 7 as well. (optional)**
   No

3. **Do you want to get recognition for this review on Publons (https://publons.com/benefits/reviewers/how)? Publons If you opt in, your Publons profile will automatically be updated to show a verified record of this review in full compliance with the journal's review policy. If you don't have a Publons profile, you will need to first create a free account. (Answer options: Yes, No)**
   No

4. **Review Comments to the Editor**

   This well-written paper is based on a well-designed study with sound data analyses. I think the paper provides interesting results and is in-line with other studies published in PLOS ONE. I have no large concerns, but do suggest some potential areas the Author could include, particularly in their Discussion; I have outlined these points in my response to the Authors. I think this paper should be accepted upon minor revisions; I would be happy to review any further submissions. Please feel free to contact me if you have questions or need clarification.

   Thank you for your patience on this review and again my apologies for any delays I have caused.
