# Supplementary material for: High nutrient loads amplify carbon cycling across California and New York coastal wetlands but with ambiguous effects on marsh integrity and sustainability
Source: PLoS One. 2022 Sep 9;17(9):e0273260. doi: 10.1371/journal.pone.0273260 (PMC9462672; doi:10.1371/journal.pone.0273260)
Supplement: Attachment — Submitted filename: Response document.docx [file pone.0273260.s005.docx]

Response to editorial and reviewer comments

Please see suggestions in normal font, and responses below in bold. I apologize if I mis-attribute some of the reviewer comments between reviewers 1-3, because they provided comments in two places: one as text and one as mark-up to documents, and it wasn't completely clear which was which.

*Editorial Office Comments*

1.	Please ensure that your manuscript meets PLOS ONE's style requirements, including

those for file naming. The PLOS ONE style templates can be found at [Link](https://journals.plos.org/plosone/s/file?id=wjVg/PLOSOne_formatting_sample_main_body.pdf) and [Link](https://journals.plos.org/plosone/s/file?id=ba62/PLOSOne_formatting_sample_title_authors_affiliations.pdf)

**I checked over that the manuscript followed the style requirements. I revised the**

**title of the Methods section to "Materials and Methods" as listed in the style guide.**

**I added a description of supplementary information. I was unable to locate any**

**information on file naming, aside from figures.**

2.	Thank you for providing information in the Ethics Statement on the field permits obtained

for this study. We ask that you additionally update your Methods section to include this

information

**I added the information on field permits and permission to the text of the**

**manuscript.**

3.	Please include your full ethics statement in the ‘Methods’ section of your manuscript file.	In your statement, please include the full name of the IRB or ethics committee who	approved or waived your study, as well as whether or not you obtained informed written	or verbal consent. If consent was waived for your study, please include this information	in your statement as well.

**I did not have an IRB or IACUC review required for this study, so there was no**

**additional ethics statements to add; I did add the information on field permits and	permission, to the methods section, as above.**

4.	We note that Figure 2 in your submission contain [map/satellite] images which may be	copyrighted. All PLOS content is published under the Creative Commons Attribution	License (CC BY 4.0), which means that the manuscript, images, and Supporting	Information files will be freely available online, and any third party is permitted to access,	download, copy, distribute, and use these materials in any way, even commercially, with	proper attribution. For these reasons, we cannot publish previously copyrighted maps or	satellite images created using proprietary data, such as Google software (Google Maps,	Street View, and Earth). For more information, see our copyright	guidelines: <http://journals.plos.org/plosone/s/licenses-and-copyright>.

We require you to either (1) present written permission from the copyright holder to	publish these figures specifically under the CC BY 4.0 license, or (2) remove the figures	from your submission:

a. You may seek permission from the original copyright holder of Figure 2 to publish	the content specifically under the CC BY 4.0 license.

We recommend that you contact the original copyright holder with the Content Permission Form (<http://journals.plos.org/plosone/s/file?id=7c09/content-permission-form.pdf>) and the following text:

“I request permission for the open-access journal PLOS ONE to publish XXX under the Creative Commons Attribution License (CCAL) CC BY 4.0 (<http://creativecommons.org/licenses/by/4.0/>). Please be aware that this license allows unrestricted use and distribution, even commercially, by third parties. Please reply and provide explicit written permission to publish XXX under a CC BY license and complete the attached form.”

Please upload the completed Content Permission Form or other proof of granted permissions as an "Other" file with your submission.

In the figure caption of the copyrighted figure, please include the following text: “Reprinted from [ref] under a CC BY license, with permission from [name of publisher], original copyright [original copyright year].”

   b. If you are unable to obtain permission from the original copyright holder to publish these figures under the CC BY 4.0 license or if the copyright holder’s requirements are incompatible with the CC BY 4.0 license, please either i) remove the figure or ii) supply a replacement figure that complies with the CC BY 4.0 license. Please check copyright information on all replacement figures and update the figure caption with source information. If applicable, please specify in the figure caption text when a figure is similar but not identical to the original image and is therefore for illustrative purposes only.

The following resources for replacing copyrighted map figures may be helpful:

USGS National Map Viewer (public domain): <http://viewer.nationalmap.gov/viewer/>

The Gateway to Astronaut Photography of Earth (public domain): <http://eol.jsc.nasa.gov/sseop/clickmap/>

Maps at the CIA (public domain): <https://www.cia.gov/library/publications/the-world-factbook/index.html> and <https://www.cia.gov/library/publications/cia-maps-publications/index.html>

NASA Earth Observatory (public domain): <http://earthobservatory.nasa.gov/>

Landsat: <http://landsat.visibleearth.nasa.gov/>

USGS EROS (Earth Resources Observatory and Science (EROS) Center) (public domain): [http://eros.usgs.gov/#](http://eros.usgs.gov/)

Natural Earth (public domain): <http://www.naturalearthdata.com/>

**I replaced the NY State aerial images with those from the public domain (USGS).**

5.	Please review your reference list to ensure that it is complete and correct. If you have

cited papers that have been retracted, please include the rationale for doing so in the	manuscript text, or remove these references and replace them with relevant current	references. Any changes to the reference list should be mentioned in the rebuttal letter	that accompanies your revised manuscript. If you need to cite a retracted article, indicate	the article’s retracted status in the References list and also include a citation and full	reference for the retraction notice.
**Changes in citations were made, but these do not include any papers that have**

**been retracted.**

- - - - - - - - - - - - - - - - - - - - - - - - - - - - - -

*Reviewer 1 Comments*

General comments

Overall I found this to be a very thorough manuscript that successfully builds the case for the research, outlines an exceptional study, effectively describes the findings in the context of the literature, and presents concluding thoughts objectively. The subject manner, the role of nutrient elevation in marsh sustainability, is incredibly important and timely. The approach of using a series of eight wetlands occurring in Atlantic (NY) and Pacific (CA) settings I found to be a highly innovative approach to enhancing breadth and applicability, and the variability in marsh settings is well detailed.

The Methods are thoroughly presented for all but the Data Analysis section, which I did find a bit lacking. If a Generalized Linear Model function was employed then I would expect there to have been at least a brief discussion of the reason underlying the selection of the technique as well as the link function employed. I'm not at all suggesting that the analysis is incorrect, just that a couple of sentences to further explain the reasoning and approach would be beneficial.

**Apologies, while we did use the glm command in R, the link function was not specified, meaning that the error structure was Gaussian, and this analysis is synonymous with OLS regression. By revising the term GLM to linear model, we think that removes the mystery as to why we chose this analysis, and did not give relevant details as to the link function employed.**

That said, I thought the use of table 2 to convey findings was effective, and the overall verbal presentation of the results was highly accessible. There were essentially no typographical errors, and I have made just a handful of suggestions to potentially improve the manuscript in the attached PDF, but those are not by any means mandatory and should be only used if the authors see the benefit.

Detailed comments

1.	Line 63: Replace "augments" with "increase"

**"Augments" was replaced with "increases," as suggested.**

2.	Line 66: Replace "increase in" with "enhanced"

**The word "enhanced" was substituted for "increase in," as suggested.**

3.	Line 157: Replace "which" with "that"

**"That" was substituted for "which" as suggested**

4.	Line 166 - add comma

**Comma was added to the sentence as suggested.**

5.	Line 261: If describing a lack of correlation, then it may be more optimal to give the	Pearson correlation rather than the R^2 value here

**The revision made as suggested.**

6.	Line 311 - add "then porewater was"

**Revision was made as requested.**

7.	Line 314 - Replace "plus" with "+"

**The word plus was replaced with the symbol "+" as requested.**

8.	Line 490 - I think the underline here is superfluous.

**The underline was removed.**

- - - - - - - - - - - - - - - - - - - - - - - - - - - - - -

*Reviewer 2 Comments*

General comments

This manuscript presents identical physical, chemical, and biological parameters associated with eutrophication, measured in contrasting marsh types on the East and West coasts of the U.S. in order to shed light on the general concept of the role of eutrophication in marsh integrity and sustainability. They do a thorough and exhaustive job of pursuing numerous interactions and explaining in detail the reasoning behind the choice of parameters to measure and how these interactions affect each other and a negative or positive effect on marsh sustainability. The authors' conclusion of ambiguity of the final results sheds light on the difficulty in assigning a definitive effect of eutrophication in all types of marsh under various conditions.

The manuscript is well written and the data is sound. I have attached a tracked changes version of the manuscript with minor typos corrected and grammatical changes. Below I repeat some comments in the margin.

1.	Lines 132-137: These two sentences may explain the main stressors to past, current,	and future wetland loss, possibly over-riding eutrophication. These factors, especially the	increased tidal range due to dredging, should be repeated at the end of the discussion	and in the conclusions as drivers of continued wetland loss, since eutrophication has	ambiguous results as the title suggests.

**An additional paragraph was added to the discussion, at the end of the 'Effects of	nutrient exposure' section to address this comment. In addition, a sentence was	added to the Conclusion paragraph: "** **We conclude that while high nutrient loads	may have negative effects on CA and NY coastal wetlands, other stressors such	as dredging which has enhanced tidal flooding, likely also play important roles."**

2.	Lines 381-383: Delete this sentence from the figure caption. It can be inserted into the	text of the manuscript, perhaps as the last sentence in the above paragraph (line 378) or	the last sentence in this section (line 395).

**The sentence was deleted; it is largely synonymous with the topic sentence of the	previous paragraph.**

3.	Lines 597-600: As with the Figure 4 caption, these 2 sentences are better moved to the	text in the conclusions, perhaps at line 581 after “(Fig 6).”, or elsewhere in the	paragraph.

**The sentence was deleted.**

Detailed comments

1.	Line 96 - Remove 'and'

**Revision made as suggested.**

2.	Line 177 - Remove dash from open space

**Revision made as suggested.**

3.	Line 193 - Delete "the"

**Revision made as suggested.**

4.	Line 206- Add dash for phrase "mid-marsh"

**Revision made as suggested.**

5.	Line 235 - Replace "at" with "per"

**Revision made as suggested.**

6.	Line 249 - Replace 1.25 year with 1.25-year

**Revision made as suggested.**

7.	Line 278 - Remove "s" from lignocelluloses

**Revision made as suggested.**

8.	Line 297 - Remove blank space.

**Revision made as suggested.**

9.	Line 298 - add comma

**Revision made as suggested.**

10.	Line 307 - add comma

**Revision made as suggested.**

11.	Line 310 - delete space

**Revision made as suggested.**

12.	Line 350 - delete four spaces

**Revision made as suggested.**

13.	Line 413 - delete "mud or"

**Revision made as suggested.**

14.	Line 425 - delete misplaced closing parentheses.

**Revision made as suggested.**

15.	Line 595 - Add dash for low-density

**Revision made as suggested.**

- - - - - - - - - - - - - - - - - - - - - - - - - - - - - -

Reviewer #3: General comments:

The Authors have a well written manuscript that outlines a study that measured a suite of belowground characteristics across salt marshes of varied nutrient exposure. The Authors noted salt marsh “drowning” in areas of high nutrient exposure, which inspired this study to determine how high nutrient exposure may influence belowground characteristics that would ultimately lead to marsh loss. The Authors sampled two sites within four estuaries, two within New York and two within California, for a total of eight salt marsh sites that ranged a nutrient exposure gradient. The Authors measured factors such as litter breakdown, belowground productivity via in-growth bags, soil respiration and nutrient content, and soil microbial biomass. The Authors found sites with higher nutrient exposure had higher belowground productivity as well as faster organic matter breakdown. Due to these results, the Authors concluded that high nutrient exposure alone could not be the cause of marsh drowning, but certainly could be a confounding factor due to the increased mineralization.

The Authors present a nicely put together manuscript. The study design, by incorporating estuaries of various nutrient exposure, cleverly uses the natural landscape to elucidate large trends to aid in our understanding of marsh stability. The data analyses and accompanying tables and figures are comprehensive and easily discernible. The writing is clean, clear, and concise.

1.	At Line 472, there is mention that soil texture can influence nutrient impacts. It would be	good to not only summarize the previous work that is referenced on how soil texture can	influence nutrient impacts but also apply this information to the study results. The	California and New York sites varied in soil texture (e.g., New York had much higher	sand content) and since California and New York sites are not evenly distributed along	the nutrient exposure gradient (e.g., California sites are the two highest nutrient	exposure sites) the soil texture differences may have played a part in findings beyond	just the nutrient exposure gradient.

**Additional discussion of soil texture was added, as recommended by the reviewer.**

2.	The Discussion would also benefit on some discussion of the differences between the	two dominant plant types used in this study. In the Results, there is mention of Salicornia	pacifica “woody nature” (Line 375-377), but this should be mentioned again in the	Discussion, along with the differences in breakdown rates between succulent forb and	graminoid leaves.

**In response to this comment (as well as the specific comment #20, referring to	Lines 475-483), a sentence was added that described the difference in	decomposition rates in response to the different C/N ratios of the two plant	species. We did not add additional information, however, as the differences	between the two species growth and turnover was covered in the third paragraph	of the 'Regional Differences' section of the discussion. If the reviewer looks over**

**this paragraph, and still feels that additional information is needed, we would be	happy to accommodate.**

Detailed comments

1.	Line 65-70: Consider adding more salt marsh related examples.

**I decided not to add specific references to salt marshes, as the following**

**paragraphs focus exclusively on salt marshes.**

2.	Line 82: Appears two spaces after the comma.

**The extra space was removed.**

3.	Line 85-86: Sentence is a bit awkwardly worded; consider revising.

**Revised from, "*Aside from coastal areas where sediments are carbonate-rich and***

***where	phosphorus is bound by calcite, coastal wetland plant communities are***

***generally nitrogen-limited,*" to "*Except in locations with carbonate soils and***

***phosphorus is bound by calcite, coastal wetland plant communities are generally***

***nitrogen-limited* "**

4.	Line 91-93: Briefly articulate how “decomposition is problematic…to wetland integrity.”

**"*Wetland integrity*" was revised to "*resilience to sea level rise*," which I believe is**

**less vague.**

5.	Line 96: Consider revising or including an example of what is meant in terms of “soil

integrity.”

**"*Soil integrity*" was revised to "*soil strength."***

6.	Line 102-105: Revise and make more concise.

**The sentence was revised from: "*In addition to this need for more information to	guide nutrient regulation and management, the recent policy emphasis on the	greenhouse gas mitigation value of wetland carbon sequestration (37) calls for	more definition around potential benefits of nutrient reductions for soil carbon	stocks."* to "*Additionally, the greenhouse gas mitigation value of wetland carbon	sequestration (37) calls for more definition around potential benefits of nutrient	reductions for soil carbon stocks."***

7.	Line 141-142: Suggest including the other two estuaries studied.

**The reference to specific estuaries was removed.**

8.	Line 145: Define what soil integrity means in the context of this study, especially as to

differentiate from earlier mentioned marsh integrity, which soil integrity appears to one of

its measures.

**Here, "marsh integrity" was revised to "marsh soil integrity." Secondly, a	definition of soil integrity was included: "Soil integrity - or the capacity of soil to	hold together and resist erosion (O'Brien 2020)..."**

9.	Line 166: What is exactly meant by “nutrient availability conditions”? Is this related to the	“nutrient exposure” that is stated later?

**Here "*nutrient availability conditions*" was revised to "*nutrient exposure*."**

10.	Line 169-172: Would be nice to re-state which two are the eutrophic estuaries. Although

it would appear that really one site in each of the two CA estuaries are eutrophic and two

are not. This may need to be stated as from the Introduction, it would seem that the

entire Elkhorn Slough estuary is considered eutrophic. Additionally, are there any signs

of marsh drowning in the other two estuaries stated here, like the two mentioned in the

Introduction?

**Additional sentences were added to the paragraph to identify the nutrient	exposure of the eight sites, as well as whether marsh loss had been documented.**

11.	Line 200: Data were not available

**The revision from** **"*data was..*." to "*data were...*" was completed as suggested.**

12.	Line 231: State that live and dead material were not separated (information inferred from

Line 510-511).

**The sentence: "*Live and dead material were not separated*." was added between**

**the last and penultimate sentence of the paragraph.**

13.	Line 238: How was material dried prior to deployment?

**An addition was made to specify the temperature the plants were dried at.**

14.	Line 245: What were the intervals that the bag collections occurred?

**A specification that litter bags were collected every two months was added.**

15.	Line 260: Soil surface temperatures?

**Soil temperatures were measured with a probe at 5-cm of depth. The specification	of the depth the soil temperature was measured at was added.**

16.	Line 311-313: Consider sentence revision as reads a bit confusing.

**Sentence was revised from, "*Porewater salinity and pH were measured, then	porewater was filtered, sub-sampled, and preserved (1:1) with a 0.22% solution of	zinc acetate (Zn(O_2_CCH_3_)_2_) for hydrogen sulfide analysis.*" to *After collection,	porewater salinity and pH were measured. Porewater was then sub-sampled, and	preserved (1:1) with a 0.22% solution of zinc acetate (Zn(O_2_CCH_3_)_2_) for hydrogen	sulfide analysis*"**

17.	Line 322-325: Consider sentence revision.

**Sentence was separated into two sentences and edited. Sentence was revised	from, "*Flooding affects oxygen availability and therefore carbon mineralization in	wetland soils, as water fills void spaces in soil, such that oxygen must diffuse	through water at a rate that is significantly slower (8400 times) than through air,	resulting in depletion of oxygen where metabolism consumes oxygen more	quickly than it can be replenished (89)." to "*** ***Flooding affects oxygen availability	and therefore carbon mineralization in wetland soils, as water fills void spaces in	soil, such that oxygen must diffuse through water at a rate that is significantly	slower (8400 times) than through air. This results in a depletion of oxygen where	metabolism consumes oxygen more quickly than it can be replenished (89). "***

18.	Line 455: “Here” isn’t needed.

**The word "here," was removed as suggested.**

19.	Line 466-469: Consider sentence revisions as reads a bit confusing.

**Sentence was revised from, "*Modeling SOM inventories provided some insight***

***into this observation, as the disequilibrium between production and	decomposition did not scale with nutrient exposure, even though both production	and decomposition did scale with nutrient exposure*," to "*While both production	and decomposition did scale with nutrient exposure, the disequilibrium between	production and decomposition did not.*"**

20.	Line 475-483: This paragraph acknowledges an important aspect and is appreciated.

Perhaps here too can add the differences in nutrient ratios between succulent and	graminoid leaves.

**Here, a sentence was added to focus on the nutrient ratios between the succulent**

**and graminoid and how that may related to the rate of decomposition. "** **However,	we did observe more rapid rates of belowground decomposition for *Salicornia*	than *Spartina* (17%), which had a lower molar CN ratio (36 vs. 41). "**

21.	Line 518-522: This should be expanded a bit more and as just stating “substrate plays

an extremely important role” doesn’t seem to fully flesh out why the results were not as

expected.

**This paragraph was revised and a sentence added to provide additional clarity.	While we can't be certain the exact reason for the results we had, consistent	relationships between microbial biomass and soil organic matter do provide a	strong explanation.**

22.	Line 591-594: Suggest also including how these regional differences may help to

understand impacts to belowground metrics.

**This phrase was added to the sentence, as suggested by the reviewer.**

23.	Figure 4: It is unclear why New York and California sites are separated here, but the

rest of the analyses compare all sites together along nutrient exposure gradient. In	addition, the colors are a bit hard to see.

**Amongst the different figures, the points are separated where there were	differences between sites (see Table 2). So for instance, there were differences	between California and New York for belowground production, so they have	separate plots. The leaf and root biomass decomposed at different rates, so they	have different plots, etc. In response to the comment about the color, we made the	color darker for the belowground production and leaf decomposition.**
